# Nanotitania Exposure Causes Alterations in Physiological, Nutritional and Stress Responses in Tomato (*Solanum lycopersicum*)

**DOI:** 10.3389/fpls.2017.00633

**Published:** 2017-04-21

**Authors:** Manish Tiwari, Nilesh C. Sharma, Paul Fleischmann, Jauan Burbage, Perumal Venkatachalam, Shivendra V. Sahi

**Affiliations:** ^1^Department of Biology, Western Kentucky University, Bowling GreenKY, USA; ^2^Department of Biotechnology, Periyar UniversitySalem, India

**Keywords:** nanoparticles, TiO_2_, tomato, redox, GST, antioxidant enzyme

## Abstract

Titanium dioxide nanoparticles (nanotitania: TiO_2_NPs) are used in a wide range of consumer products, paints, sunscreens, and cosmetics. The increased applications lead to the subsequent release of nanomaterials in environment that could affect the plant productivity. However, few studies have been performed to determine the overall effects of TiO_2_NPs on edible crops. We treated tomato plants with 0.5, 1, 2, and 4 g/L TiO_2_NPs in a hydroponic system for 2 weeks and examined physiological, biochemical, and molecular changes. The dual response was observed on growth and photosynthetic ability of plants depending on TiO_2_NPs concentrations. Low concentrations (0.5–2 g/L) of TiO_2_NPs boosted growth by approximately 50% and caused significant increase in photosynthetic parameters such as quantum yield, performance index, and total chlorophyll content as well as induced expression of *PSI* gene with respect to untreated plants. The high concentration (4 g/L) affected these parameters in negative manner. The catalase and peroxidase activities were also elevated in the exposed plants in a dose-dependent manner. Likewise, exposed plants exhibited increased expressions of glutathione synthase and glutathione *S*-transferase (nearly threefold increase in both roots and leaves), indicating a promising role of thiols in detoxification of TiO_2_NPs in tomato. The elemental analysis of tissues performed at 0.5, 1, and 2 g/L TiO_2_NPs indicates that TiO_2_NPs transport significantly affected the distribution of essential elements (P, S, Mg, and Fe) in roots and leaves displaying about threefold increases in P and 25% decrease in Fe contents. This study presents the mechanistic basis for the differential responses of titanium nanoparticles in tomato, and calls for a cautious approach for the application of nanomaterials in agriculture.

**GRAPHICAL ABSTRACT:**
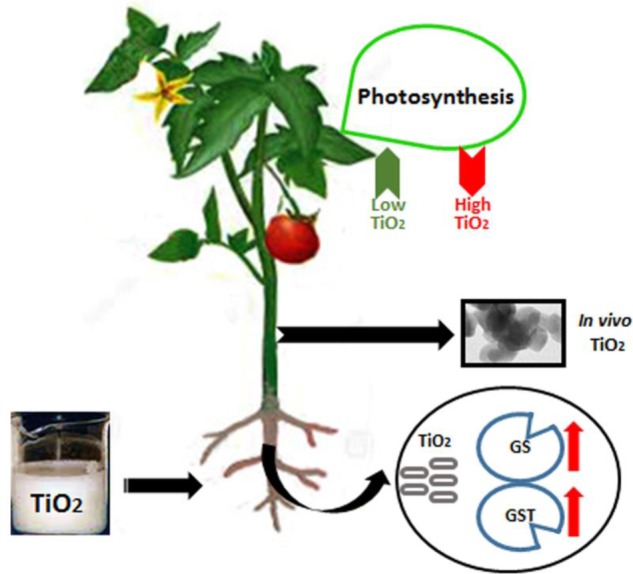
**Movement of nanotitania in plant tissues**.

**Movement of nanotitania in plant tissues**.

## Introduction

In the recent past, technological breakthrough in nanoengineering coupled with the recognition of nanotechnology potential led to a rapid application of nanomaterials in a variety of ways including appliances, electronics, food, health, agriculture, and health fitness (Chen and Mao, 2007). The proportion of titanium dioxide nanoparticles (TiO_2_NPs) tops the list of nanomaterials commercially used these days (Piccinno et al., 2012). Basically, TiO_2_NPs are synthesized in three forms namely anatase, rutile, and brookite with individual characteristics. Anatase is structurally a dipyramidal tetragon and typically used in photocatalytic applications whereas rutile, a prismatic tetragon, used primarily in paints and other consumer products. Brookite has an orthorhombic structure and is also used in photocatalytic applications (Macwan et al., 2011). TiO_2_NPs also serve as a common food additive used to enhance the color, brightness, and sometimes flavor of a variety of food products (Peters et al., 2014).

Given the diverse applications of TiO_2_NPs in multiple sectors, the demand of TiO_2_ is consistently rising and; according to a report, 7,800–38,000 tons/year TiO_2_NPs were produced only in US (Hendren et al., 2011). Due to widespread use, TiO_2_NPs easily enter into the environment via direct discharge or through water channeling systems. The deliverable proportion of TiO_2_NPs-containing cosmetics into the environment is the highest among others, and accounts for 70–80% of nanotitania alone (Piccinno et al., 2012). High concentrations of these nanomaterials are also found in the downstream of wastewater treatment plants. Titanium nanomaterial removal and release from wastewater treatment plants was recently measured by Kiser et al. (2009). This study indicates that raw sewage contains 100–3000 μg/L of Ti, while its concentration in wastewater effluents ranges in 5–15 mg/L. When removed, Ti accumulates in biosolids with concentrations varying from 1 to 6 g/kg (Menard et al., 2011). The effect of TiO_2_ nanoparticles on local land flora and fauna, after land application in the form of biosolids, or water bodies is poorly understood, and need to be evaluated.

The occurrence of high TiO_2_ in soil and water bodies encouraged researchers to examine the associated impact on various life forms in recent times. A number of studies have revealed the toxicity effects of TiO_2_NPs on microorganisms, invertebrates, and higher plants (Menard et al., 2011). The toxicity assay using soil bacteria *Pseudomonas putida* and microalga *Pseudokirchneriella subcapita* demonstrated that TiO_2_NPs severely affect the growth and viability of these microorganisms (Aruoja et al., 2009; Combarros et al., 2016). A study indicated that TiO_2_ toxicity in marine microalga, *Nitzschia closterium*, mainly arises from an increased reactive oxygen species (ROS) level (Xia et al., 2015). Current studies reveal how metal oxide engineered nanoparticles enter through plant root or leaves, move upward or downward, and accumulate in aerial fruits or underground tubers (Bradfield et al., 2017; Tripathi et al., 2017). Significant phytotoxic effects of TiO_2_NPs were reported in higher plants such as *Ulmus elongate* (Gao et al., 2013), *Triticum* sp. (Larue et al., 2012), *Hordeum vulgare* (Mattiello et al., 2015), and *Zea mays* (Morteza et al., 2013). The range of toxic effects include inhibition of seed germination, seedling elongation, photosynthetic efficiency of shoot, generation of ROS, cytotoxicity and genotoxicity (Cox et al., 2016; Du et al., 2017). Genome-wide expression analysis of the effect of nanoparticles including TiO_2_ has shown that the root hair development and antistress defense responses are significantly affected in *Arabidopsis* (Garcia-Sanchez et al., 2015). TiO_2_NP exposure repressed the gene expressions related with pathogen defense, and, thereby, increased the bacterial colonization in rhizosphere (Garcia-Sanchez et al., 2015). The nZnO, nTiO_2_-induced transcriptome analysis in *Arabidopsis* showed the altered expression of genes mainly involved in biotic and abiotic stimuli (Landa et al., 2012). This study further suggests that the mechanisms of nanoparticles toxicity in plants are specific to the metal type, dimension and surface characteristics of nanoparticles despite some overlaps in gene expression patterns (Landa et al., 2012). A similar transcriptome-based study in *Arabidopsis* also points to a host of differentially expressed genes induced by silver nanoparticles (AgNPs), besides an implication of Ag^+^ in causing stress responses in plants (Kaveh et al., 2013). The treatment of different concentrations of TiO_2_NPs induced phytotoxic effects, such as, ROS production and DNA damage in *Vicia narbonensis* (Ruffini Castiglione et al., 2014).

Tomato (*Solanum lycopersicum*) is an important edible crop growing worldwide including in USA, California, and Florida being the leading producers. The high TiO_2_NPs content in irrigation water led to conduct few toxicological investigations on tomato. The functional analyses of uptake and translocation of TiO_2_NPs in different plant parts of tomato revealed a differential accumulation of these nanomaterials in these tissues (Song et al., 2013a; Raliya et al., 2015). However, these studies are insufficient to understand the mechanistic and molecular process associated with TiO_2_NPs toxicity. In the present investigation, we have studied the effect of TiO_2_NPs on growth of tomato using multi-pronged approaches: physiological, biochemical, and molecular. Our objectives were to study (i) plant biomass increase, (ii) photosynthetic efficiency, (iii) role of tissue accumulation of TiO_2_NPs in elemental nutrition, (iv) antioxidative enzyme activities, and (v) role of glutathione in detoxification – under the exposure of TiO_2_NPs. This study demonstrates the growth stimulatory role of TiO_2_NPs under lower doses while growth inhibitory roles under higher doses, and further establishes a promising role of thiols in cellular detoxification against nanotitania.

## Materials and Methods

### Plant Materials and Growth Conditions

*Solanum lycopersicum* was used as plant material in this study. Seeds (Five Star Grape, F1) were procured from Johnny’s Seed Co. (USA). Tomato seeds were surface sterilized as indicated by Tiwari et al. (2014) and stratified in sterile nanopure water for 48 h at 4°C. After stratification, seeds were placed in trays containing sterile soilrite soaked with half strength Hoagland solution. Seeds were grown in a Percival growth chamber under controlled light, temperature, and humidity. The growth chamber was maintained at 25°C ± 2°C temperature, 16/8 light/dark photoperiod cycle and 130–180 μmol m^-2^s^-1^ of light intensity. After 2 weeks of growth, 8–10 plants of the same height for each group were collected; roots were washed thoroughly, and transferred to hydroponic setup maintained in Magenta boxes containing half strength Hoagland solution.

### TiO_2_ Nanoparticles Treatment

After 3 days of growth in Hoagland solution, the solution was replaced with four concentrations of rutile titanium dioxide nanoparticles (0.5, 1, 2, and 4 g/L) diluted in deionized water. The group filled with only water served as control. The colloidal TiO_2_NPs solution was purchased from US Research Nanomaterials, Inc. (Product No. US 7070). According to manufacturer’s description, the TiO_2_NPs were 30–50 nanometers in size with 99.9% purity. Size range was also verified by transmission electron microscopy (TEM) analysis in this study. Plants were allowed to grow for the uptake of TiO_2_NPs for 4 days with regular agitation, and then again replaced with Hoagland solution for 3 days. The cycle was repeated for one more week. Such repetition was performed in order to make sure that plants were not deprived with nutrients. Treatments were run for 2 weeks before they were harvested for the different estimations.

### Biomass Estimation

For biomass measurement, whole plants were harvested after completion of experiment and rinsed with deionized water. The plants were dried in an oven at 60°C for 2 days, and then weighed to determine dry weight. Biomass was calculated by the average dry weight of replicates in each treatment group.

### Measurement of Photosynthetic Efficiency

The photosystem efficiency was estimated by measuring chlorophyll fluorescence using a Hansatech Instruments Handy-PEA chlorophyll fluorimeter. After the treatment, plants were allowed to adapt in dark for a minimum of 30 min before excitation of 1 s pulse of red light. The following parameters were measured using the Handy-PEA: *F*_0_ (minimum chlorophyll fluorescence after excitation), and *F*_m_ (maximum chlorophyll fluorescence after excitation). These two parameters were used to calculate *F*_v_ (variation in fluorescence calculated from *F*_0_ and *F*_m_), *F*_v_/*F*_m_ (ratio of variable fluorescence and maximum fluorescence), and *F*_v_/*F*_0_ (ratio of variable fluorescence and minimum fluorescence).

### Chlorophyll Estimation

For the chlorophyll estimation, 100 mg of leaf tissue was harvested and placed in 10 ml of 70% ethanol. After incubating at room temperature for 3 days, the absorbance of supernatant was measured at 648.6 and 664.2 nm wavelength. The final concentration was calculated according to the method described by Lichtenthaler (1987).

### Antioxidative Stress Enzyme Assay

The crude enzyme was extracted by following the method described by earlier (Hamurcu et al., 2013). Briefly, 250 mg of frozen tissue was fine ground in liquid nitrogen and mixed with 10 ml of sodium phosphate extraction buffer. The extract was then centrifuged at 4°C at 12000 ×*g* for 20 min. The supernatant was collected in separate tube and protein was quantified using Bradford method (Bradford, 1976).

Catalase (EC 1.11.1.6) activity was determined using the earlier described method (Gusman et al., 2013). The 0.1 ml of crude enzyme extract were added to 2.9 ml of potassium phosphate buffer, pH 7.0, containing 12.5 mM H_2_O_2_. The mixture was incubated for 1 min at 30°C and the absorbance was measured at 240 nm. Peroxidase (EC 1.11.1.7) activity was determined by adding 0.1 ml of crude enzyme extract into 4.9 ml of potassium phosphate buffer containing 20 mM pyrogallol and 20 mM H_2_O_2_ (Kar and Mishra, 1976). The absorbance was measured at 420 nm after 1 min of reaction. The activity was calculated using the molar extinction coefficient 420 nm; έ 2.47 mM^-1^ cm^-1^ (Gusman et al., 2013).

### Scanning Electron Microscopy

Initially roots were frozen, fractured in liquid nitrogen. The freeze-fractured samples were kept at −20°C for 20 h in lyophilizer, and then mounted onto aluminum stubs. Samples were then examined at an accelerating potential of 20 kV under high vacuum mode with the backscatter detector in place using a JSM-5400LV scanning electron microscope (SEM) (JEOL) equipped with IXRF EDS system with a Moxtek AP3.3 light element entrance window. The spotted nanoparticles region were confirmed by EDX spectra analysis.

### Quantitative Real Time PCR Analysis

Total RNA was isolated from plant replicates under TiO_2_NPs treatments (up to 2 g/L) using Spectrum Plant Total RNA Kit (Sigma). Two microgram RNA was subjected to RQ1 DNase (Promega) and reverse transcribed using the SuperScript III first-strand cDNA synthesis kit (Invitrogen). The quantitative RT-PCR reaction was performed in 10 μl total volume that contains 5 μl of 2X SYBR (ABI Biosystems, USA), 1 μl of five times diluted cDNA, 1 μl of forward and reverse primer (5 μM), and 2 μl nuclease free water. The thermal cycling conditions were: 10 min at 95°C for initial polymerase activation, 15 s at 95°C for denaturation, 1 min at 60°C for anneal/extension, and 40 cycles in a 96-well reaction plate in Applied Biosystems 7300 thermal cycler (Applied Biosystems, USA). To normalize the expression data, ubiquitin was used as an endogenous control and the relative expression levels of the gene was calculated by 2^-ΔΔC_t_^ method (Livak and Schmittgen, 2001). The gene specific primers were designed using NCBI primer design tool. The details of primer sequences are listed in **Supplementary Table S1**.

### Metal Estimation

Root, stem and leaves from plant replicates under TiO_2_NPs treatments (up to 2 g/L) were separated and thoroughly washed with deionized water at least 10 times. The root and leaf samples were kept in desorption solution (2 mM CaSO4 and 10 mM ethylenediaminetetraacetic acid) for 10 min (Tiwari et al., 2014). After three times further washing, samples were dried at 65°C for heat drying for 5 days. Dried plant tissues (25–30 mg) were digested in 5 ml of concentrated nitric acid at 80°C for 3 days. All samples were three times diluted in nanopure water (Tiwari et al., 2016). Elemental analysis was carried out by Inductively Coupled Plasma Emission Spectroscopy (ICP-ES).

### Statistical Analysis

Treatment and control means were analyzed by one-way analyses of variance using SYSTAT (Version 12 for Windows, 1999, Systat Software Inc., Richmond, CA, USA). Where variance ratios were significant between treatments (*p* < 0.05), they were compared using LSD (*P* = 0.05) for each treatment separately.

## Results and Discussion

### A Differential Effect of TiO_2_NPs on Tomato Plant Growth

**FIGURE 1:**
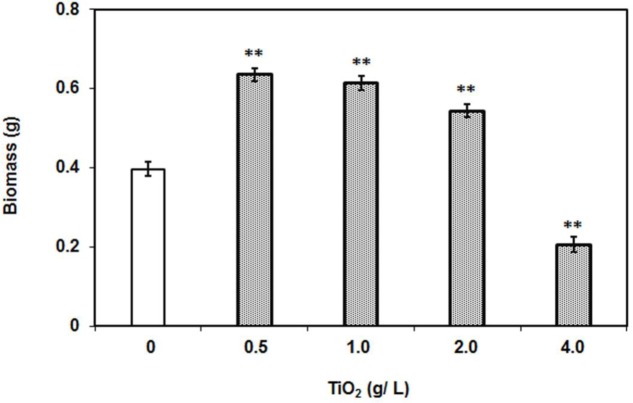
**Dry weight of individual plants after 2 weeks of TiO_2_NPs exposure.** The bars represent means ± SEM (*n* = 10–14). Asterisk (^∗∗^) denotes significant difference (*P* > 0.001) compared to control (no TiO_2_NPs).

Biomass measurement is a key attribute for studying the behavior of plant in response to metal and abiotic stresses on overall performance. To investigate the effect of TiO_2_ nanoparticles, 2 weeks old tomato plants were subjected to increasing concentrations of TiO_2_NPs and allowed to grow for further 2 weeks. Interestingly, data indicated that lower concentration of TiO_2_NPs favored the plant growth and increased the total biomass (**Figure 1**). The biomass at 0.5 g/L TiO_2_NPs was 50% greater than control. Thereafter, a successive reduction in biomass occurred on increasing concentrations (1 and 2 g/L TiO_2_), yet biomass increase remained significantly greater than in control. Nevertheless, high dose of 4 g TiO_2_NPs/L was found to be toxic affecting seedling growth (about 50% reduction with respect to control: **Figure 1** and Supplementary Figure S1). At this concentration, the total biomass of plants significantly reduced (52%) with respect to control. Consistent to our results, an earlier study had demonstrated that critical TiO_2_NPs concentrations enhanced the growth and development of tomato (Raliya et al., 2015). The application of TiO_2_NPs was found to increase the root length of other plants such as rice (Wang et al., 2015). Similar response was observed in *Arabidopsis* where root elongation occurred in response to low concentrations of rare earth metal nanoparticles, while root growth inhibition followed at higher concentrations of those nanoparticles (Ma et al., 2013). The root growth inhibition at high concentrations of nanoparticles could be due to higher acquisition of nanoparticles in root cells. Another study reported a contrasting observation where TiO_2_NPs had not affected significantly the growth of oilseed rape, lettuce, and kidney bean at the same concentration used in this study (Song et al., 2013b). From perusal of toxicity studies, it appears that plant response to nanomaterials is species-dependent.

### TiO_2_NPs Alter Photosynthetic Efficiency in Tomato Plant

**FIGURE 2:**
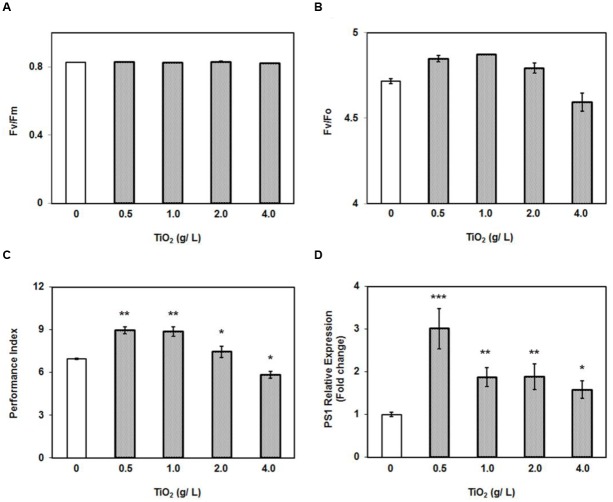
**Effect of 0–4 g/L TiO_2_NPs on photosynthetic efficiency of tomato. (A)** *F*_v_/*F*_m_ ratio. **(B)** *F*_v_/*F*_o_ ratio. **(C)** Performance index of plants grown under same conditions. **(D)** Quantitative RT-PCR analysis of *PS1*. Bars represent means ± SEM (*n* = 10–14) where significant difference are represented by asterisks on the top of respective bars.

The growth stimulating effect of low concentrations of TiO_2_NPs on tomato plants encouraged us to measure the photosynthetic efficiency. The photosynthetic parameters were studied in various groups of plants under nanoparticle exposure. There was no change observed in maximum quantum yield of the photosystem (*F*_v_/*F*_m_) under different treatments (**Figure 2A**). Numerous reports have showed that *F*_v_/*F*_m_ ratio is constant at certain abiotic stresses (Baker and Rosenqvist, 2004; Baker, 2008). However, the ratio between the variable to minimal fluorescence (*F*_v_/*F*_o_) in treatment groups, except 4 g/L, was significantly greater than in control and corroborated with the pattern in biomass increase (**Figures 1**, **2B**). It should be noted that *F*_v_/*F*_o_, unlike *F*_v_/*F*_m_, is non-linearly related to the maximum quantum efficiency of photosystem II (Baker and Rosenqvist, 2004). The *F*_v_/*F*_o_ ratio reflects contribution of the electron trapping potential which is normally referred to as quantum yield in photochemistry and indicative of overall performance of plant. Similarly another important parameter, performance index (PI_ABS_) was also recorded highest at 0.5 g/L TiO_2_NPs, and which subsequently decreased with rise in the concentration of nanotitania, but yet maintaining significantly higher levels than in control (**Figure 2C**). Again, PI_ABS_ bottomed under the treatment of 4 g/L TiO_2_NPs, being significantly lower than control. In general, PI_ABS_ is a multi-parametric quantity that indicates the performance of photosystem II (PSII), for instance, trapping of excitation energy, electron transport and dissipation of excess excitation energy. PI_ABS_ seems to be a suitable and sensitive parameter to investigate comprehensive photosynthetic performance of plant under different abiotic and biotic stresses (Strasser et al., 2000). It represents the functionality of photosystems I and II in the quantitative way which ultimately manifests the plant performance under different stress conditions (Strasser et al., 2004).

Moreover, in order to corroborate photosynthetic observations at molecular level, we measured the expression of *PSI* gene (*P700 apoprotein A2*), which constitutes the integral part of photosystem I. The RT-PCR data clearly demonstrated that *PSI* was significantly induced at all the concentrations of TiO2NPs studied (**Figure 2D**). An interesting trend observed was the highest expression of *PSI* gene at 0.5 g/L TiO_2_NPs. Unquestionably, TiO_2_NPs at 0.5 g/L appeared to be an optimal concentration supporting growth and photosynthetic efficiency including expression of *PSI*. It has been shown that TiO_2_NPs could induce the light harvest complex II (LHCII) gene expression in *Arabidopsis*, promote the light absorption of chloroplast, and regulate the light distribution between photosystem I and photosystem II by increasing LHCII activity (Ze et al., 2011). Therefore, growth promoting potential of lower dose of TiO_2_NPs appears to have resulted by accelerating the photosynthetic efficiency of tomato plants.

**FIGURE 3:**
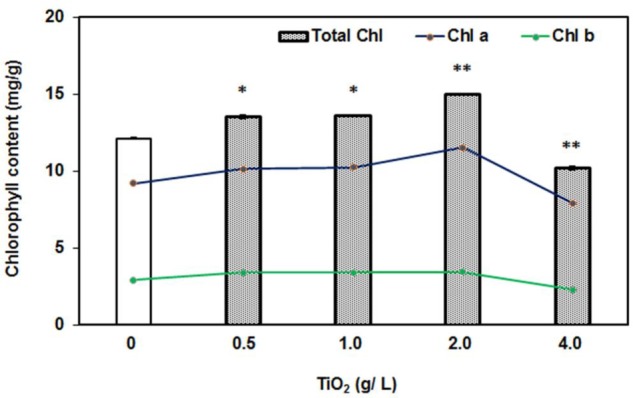
**Effect of 0–4 g/L TiO_2_NPs on chlorophyll content after 2 weeks of exposure.** Bars represent total chlorophyll quantity, whereas lines are representative of quantities of Chl *a* and Chl *b*. An increase of 50% in the total chlorophyll content in low doses, while 50% decrease with respect to control in case of 4 g/L can be noted. Single asterisk indicates the significant difference (*P* > 0.01) from contol while double asterisks indicate significant differences from control and first two concentrations.

Furthermore, we also measured the chlorophyll content in the leaves under exposure of TiO_2_NPs concentrations. It was observed that total chlorophyll content had remarkably increased with respect to control under the treatment of 0.5–2.0 g/L TiO_2_NPs (**Figure 3**). Similar patterns were observed with foliar application of TiO_2_NPs on *Z. mays* in which case it was thought to be a promising way to increase crop yield (Morteza et al., 2013). However, the high concentration such as 4 g/L TiO_2_ had diminished the chlorophyll accumulation in tomato (**Figure 3**). Contents of chlorophyll a and chlorophyll b followed the same trend (**Figure 3**). These observations were supported by a number of earlier studies reporting the loss of chlorophyll content in response to cerium oxide nanoparticles in *Arabidopsis* (Ma et al., 2013), silver nanoparticles in *Spirodela polyrhiza* (Jiang et al., 2012), and green algae (Oukarroum et al., 2012). Studies have shown that nanoparticles inhibit the expression of chlorophyll synthesis genes and photosystem-related genes, which ultimately lead to the reduction in photosynthesis efficiency in the plants (Navarro et al., 2015; Wang et al., 2016). Many aquatic organisms of order Cladocera appeared to be very sensitive to TiO_2_NPs, which caused significant phytotoxicity in these organisms at a low concentration (Jovanovic, 2015). The present investigation demonstrates the dual role of nanotitania on tomato plant: growth stimulatory up to certain low doses and growth inhibitory thereafter.

### Uptake and Transport of TiO_2_NPs by Tomato Plant

**FIGURE 4:**
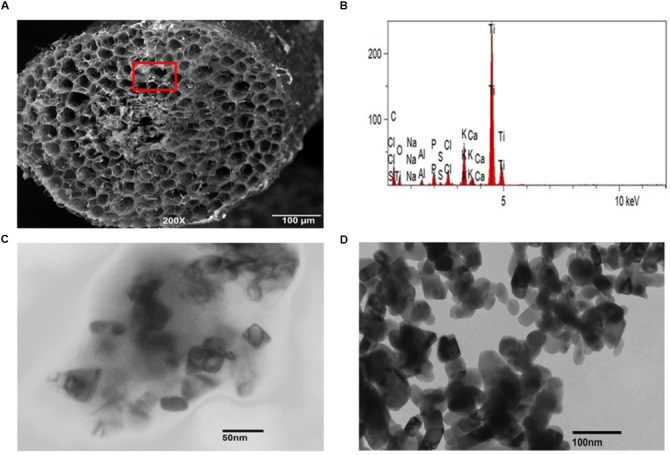
**Presence of TiO_2_NPs inside tomato tissues. (A)** Scanning electron microscope (SEM) image shows TiO_2_NPs spot in the form of white granules in the inner region of freeze-fractured root cells. **(B)** EDX spectrum of white granules spotted in cortical cells (marked with red square). The visible peaks confirmed the presence of Ti in white spots. **(C)** Transmission electron microscopy (TEM) image showing the presence of TiO_2_NPs inside tomato stem cells after 2 weeks of exposure. **(D)** TEM image showing cylindrical/tetragonal geometry of rutile TiO_2_NPs used for treatment.

The role of membrane transporters in the uptake of nutrients such as P, Cu, Fe, or Zn is well-known, but knowledge about how the inert metallic nanoparticles enter plant cells and then move to different tissues/organs is limited. To determine uptake of TiO_2_NPs by the root cells, we performed localization experiment using SEM. Freeze-fractured root tissue samples derived from treated plants were examined for the presence of TiO_2_NPs in root tissues. Evidence of TiO_2_NPs presence was observed in the cortical regions of a number of root samples (**Figure 4A**). It was further confirmed by energy dispersive X-ray spectroscopy (EDX) analysis (**Figure 4B**) and ICP-MS analysis of root (Supplementary Figure S2). The SEM coupled with EDX is considered as a reliable and method for studying the nanoparticle topography in tissues. Furthermore, to determine the transport of nanomaterials across tissues, stem parts of tomato plant were also examined by TEM. Scattered accumulations of titanium tetragonal and cylindrical crystals were found in various samples under TEM (**Figure 4D**; compared with **Figure 4C**). Previous studies on *Phaseolus vulgaris*, *Triticum aestivum*, and *Rumex crispus* have also demonstrated the transport of TiO_2_NPs across plant tissues (Jacob et al., 2013). Xia et al. (2015) reported the internalization of TiO_2_NPs into marine alga, *N. closterium* through TEM and cytometry. Earlier analyses of titanium, silver, and zinc nanoparticles uptake and toxicity revealed that these nanoparticles were accumulated in stem, leaves, and fruits of tomato and cucumber (Servin et al., 2013; Ruffini Castiglione et al., 2014; Garcia-Sanchez et al., 2015). The presence of nanomaterials was observed in cortical cells of tomato root or shoot in our investigation, but we could not detect their presence in the conducting tissues. We thus speculate that Ti nanoparticles might have moved across tissues through mass flow of water/apoplastic transport. However, studies reported that nanoparticles could be taken up by the plant through direct penetration and translocation of nanoparticles from leaf to root shows evidence that nanoparticles travel by the phloem transport mechanism (Gogos et al., 2016; Raliya et al., 2016). Despite reports of distribution of TiO_2_NPs in different plant parts, the exact transport pathway, mechanism, role of channels and transporters in translocation of nanoparticles are not mapped out.

### Accumulation of TiO_2_NPs Affects Nutritional Balance in Tomato

**FIGURE 5:**
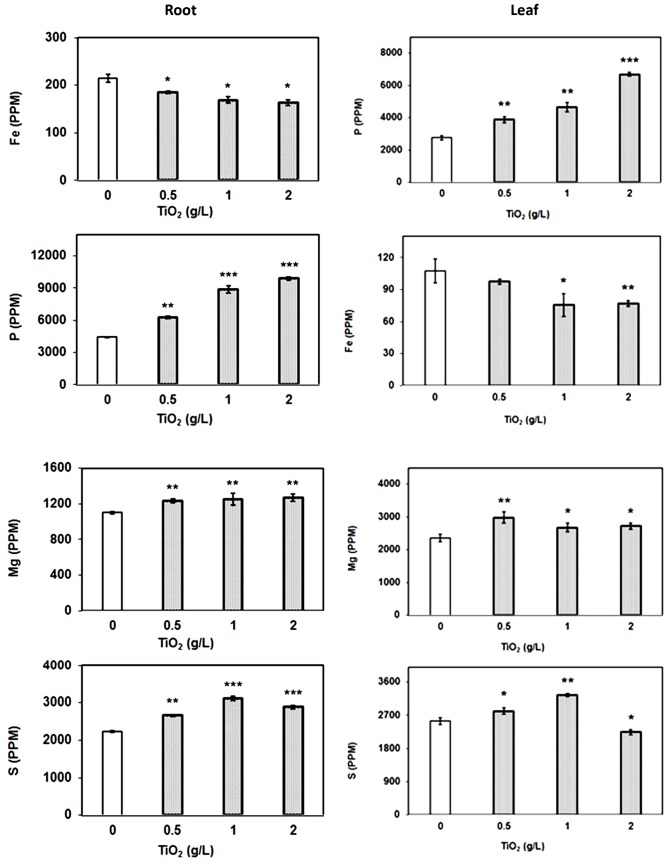
**Elemental profile of plant root and leaves.** The element analysis was carried out by ICP-MS in root and leaf samples treated with TiO_2_NPs. The data are means of three biological replicates ± SEM where significant difference are represented by asterisks on the top of respective bars.

To assess the impact of TiO_2_NPs on other essential elements, we also simultaneously carried out elemental profile by measuring the content of iron (Fe), phosphorus (P), sulphur (S), and magnesium (Mg) in root and leaf samples. The data indicated that P, S, and Mg contents were significantly increased with respect to control in both root and leaves after TiO_2_NPs treatments (**Figure 5**). It is well understood that P is a limiting nutrient for plants and it must be available in high concentrations to support increase in growth. Mg plays a critical role in photosynthesis by occupying the central atom in the formation of chlorophyll molecules, and also affecting the photophosphorylation process. Thus, TiO_2_NPs induced P and Mg uptake and further supported our photosynthetic observations. The increase of S content in response to TiO_2_NPs is understandable as it is the principle amino acid of GSH meant for detoxification of metals and xenobiotic (Jacob et al., 2013). Contrary to these elements, Fe content significantly decreased in root as well as leaves under TiO_2_NPs exposure (**Figure 5**). We suspect if Ti competes with Fe uptake from the solution. Further studies can reveal the substitution of Fe by Ti. Overall, our study indicates that the exposure of titanium nanoparticles can cause nutritional imbalance in plants affecting growth and physiology at toxic doses.

### TiO_2_NPs Evoke Antioxidative Stress Enzyme Activity

**FIGURE 6:**
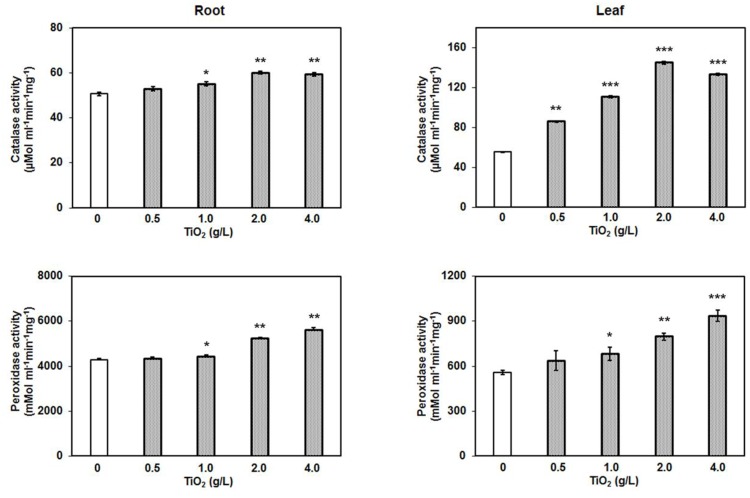
**Catalase and peroxidase assay after TiO_2_NPs exposure in root and leaf.** Bars represent means ± SEM (*n* = 3) where significant difference are represented by asterisks on the top of respective bars.

Oxidative stress is the common response of cells toward various stresses. The relationship between oxidative stress and metal or abiotic stress is well-known. The metal stress commonly generates toxic free radicles and superoxides, and to prevent the causal damage, cell activates the production of antioxidative stress enzymes such as peroxidase, catalase, ascorbate peroxidase, and superoxide dismutase. These enzymes scavenge the ROS by reduction reaction and protect cells from any damage (Gao et al., 2013; Garcia-Sanchez et al., 2015). The catalase activity in tomato root and leaf increased depending on the concentration of TiO_2_NPs. Similarly, peroxidase activity was also enhanced, displaying the maximum activity at 4 g/L TiO_2_NPs (**Figure 6**). These results are in agreement with previous studies that reported the induction of antioxidative stress enzymes activity after exposure of silver nanoparticles in plants (Cox et al., 2016). TiO_2_NPs exposure to *V. narbonensis* caused high ROS production and the surge of associated antioxidant enzyme activities (Ruffini Castiglione et al., 2014). Nanotitania has a tendency to activate antioxidant enzyme activity, for instance, peroxidase, catalase, and superoxide dismutase among different life forms ranging from marine microalgae (Dalai et al., 2014; Xia et al., 2015) to higher plant *H. vulgare* (Mattiello et al., 2015). Similar reports of operative oxidative and antioxidative activities appeared in an excellent review article published recently (Cox et al., 2016).

Results of these enzyme assays in the present study indicate that higher concentrations of TiO_2_NPs cause significant oxidative stress in tomato, eventually inducing the production of antioxidative enzymes. Considering the fact that TiO_2_NPs largely impacted the distribution of essential metals from root to shoot, it might be possible that oxidative stress in leaf was the indirect result of an alteration in other essential metals rather than TiO_2_NPs. Therefore, here we hypothesize that activation of redox related enzyme activities in leaf was the consequence of crosstalk between titanium and essential elements in root, transmitting the signal up to the shoot. Our results suggest that TiO_2_NPs induced the intracellular ROS generation that ultimately increased the catalase and peroxidase activities in order to cope with the oxidative stress, and to maintain the functional redox state in tomato up to a certain level (2 g/L).

### Glutathione Plays Important Role in Detoxification of TiO_2_NPs

Given the virtue of reversible oxidization, thiols are recognized as key component involved in the overall maintenance of redox balance. Glutathione (GSH) is a small peptide (γ-glutamylcysteinylglycine) molecule having indispensable role in biosynthetic pathways, detoxification and redox homeostasis in plant. The glutathione serves as precursor molecule for the synthesis of phytochelatins (PCs), small polypeptides with repeating γ-EC units, via enzymatic reaction catalyzed by phytochelatin synthase (Grill et al., 1989). Heavy metals mostly induce the synthesis of PCs via an activation of phytochelatin synthase and induction of glutathione synthesis in plants. Similarly, studies have indicated that nanoparticle treatments also activate the thiol synthesis in plants (Grill et al., 1989;Ma et al., 2013). Hence to study the effect of TiO_2_NPs, relative expression of genes involved in the synthesis and modification of GSH was examined.

**FIGURE 7:**
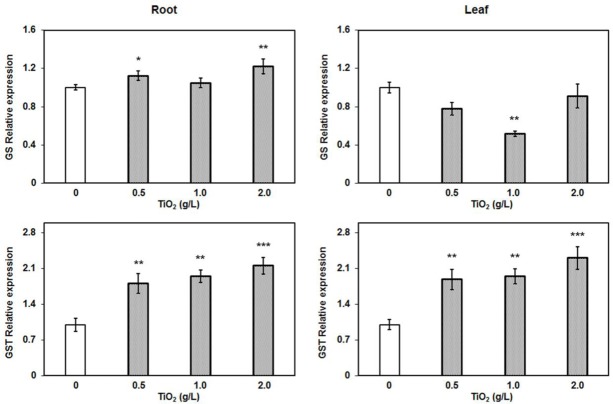
**Quantitative RT-PCR relative expression analysis of major detoxifying genes in root.** The glutathione synthase and glutathione *S*-transferase (*z*-class) expression were induced after TiO_2_NPs exposure. Data represent means ± SEM (*n* = 3) where significant difference are represented by asterisks on the top of respective bars.

The RT-PCR results revealed that glutathione synthase expression was upregulated in root in contrast to the repressed expression in leaf (**Figure 7**). The glutathione synthase catalyzes the addition of glycine with γ-glutamylcystein which is the second strep of GSH production. Furthermore, we also measured the expression of glutathione *S*-transferases (GSTs). We randomly selected three GST genes: GSTZ1, DHAR1, and GSTL1, and performed RT-PCR to detect their expressions. Among them, only the expression of GST from zeta class (GSTZ1) was significantly increased in both root and leaf tissues in a concentration dependent manner (**Figure 7**). The GSTZ1 is a member of the GST superfamily of proteins that catalyze the reaction of glutathione with various intracellular substrates and xenobiotic (Edwards and Dixon, 2005). GSTs commonly provide tolerance against a range of abiotic stresses, indicating their protective roles in plants. Unlike the phi and tau classes of GSTs, zeta GSTs act as glutathione dependent isomerases, catalyzing the conversion of maleylacetoacetate to fumarylacetoacetate, a step in tyrosine degradation. The GSTZ1 has a high degree of functional overlap and mostly involved in overcoming the cellular oxidative stresses. Therefore, upregulation of *GSTZ1* may be the result of oxidative stress during exposure of TiO_2_NPs in tomato. The cellular redox balance is regulated by multiple enzymatic systems and genetic controls that prevail overcoming toxic conditions in cells. Hence, the upregulation of *GS* and *GSTZ1*, associated with elevated S contents in root and leaves, clearly demonstrate the role of thiol-based cellular redox system in detoxification against TiO_2_NPs exposure up to 2 g/L in tomato.

Nano-toxicological studies have gained intense research interests in past few years after widespread biotechnological applications of nanomaterials in areas from food, cosmetics to medicine. The expansion of the use of TiO_2_NPs results into its potential exposure to environment. However, there is limited information to understand the eco-toxicological impact of TiO_2_NPs in important crops. This study is an effort to understand the comprehensive responses of TiO_2_NPs on crops species such as *S. lycopersicum*. We report the dual role of Ti nanoparticles on plants: low concentrations while promoting growth and overall plant health, irrespective of Ti accumulation inside plant cells, higher concentrations producing high oxidative stress and affecting growth and photosynthetic efficiency under short term exposure in hydroponic system. The reduced crop biomass, photosynthetic yield, plant performance, and potential Ti accumulations in plant fruits at higher TiO_2_NPs concentrations are serious concerns for agriculture and food. Therefore, agricultural application of Ti nanoparticles to boost productivity and quality may not be free from a risk to the ecosystems and environment.

## Author Contributions

SS and NS developed the idea and designed the experiments. MT, PF, and JB performed the experiments. MT analyzed the data, prepared the figures, and wrote the manuscript. NS rewrote and edited the manuscript. All authors reviewed the manuscript.

## Conflict of Interest Statement

The authors declare that the research was conducted in the absence of any commercial or financial relationships that could be construed as a potential conflict of interest. The reviewer BNS and handling Editor declared their shared affiliation, and the handling Editor states that the process nevertheless met the standards of a fair and objective review.

## Supplementary Material

TABLE S1 **List of primers used for different genes**.
