# Supplementary material for: Nanotitania Exposure Causes Alterations in Physiological, Nutritional and Stress Responses in Tomato (Solanum lycopersicum)
Source: Front Plant Sci. 2017 Apr 21;8:633. doi: 10.3389/fpls.2017.00633 (PMC5399031; doi:10.3389/fpls.2017.00633)

**Supporting Table**

| **S.N.** | **Primer name** | **Primer sequences (5’-3’)** |
| --- | --- | --- |
|  | PS1 F | ATAAGGGTCTTTATGACACA |
|  | PS1 R | TATAATGCAGCTTGAGTAGT |
|  | GS F | ATGGTGATCCAAGGGCTGTG |
|  | GS R | GCAGAAAGCCAATGCTGGTC |
|  | GST F | GAGAAGTATCCCCAGCGAGC |
|  | GST R | TCCTGGATGTACTTCTGATAGTTG |
|  | UBQ F | AGGTTGATGACACTGGAAAGGTT |
|  | UBQ R | AATCGCCTCCAGCCTTGTTGTA |

**Figure S1:** Effect of different concentrations of TiO_2_ on growth of tomato.


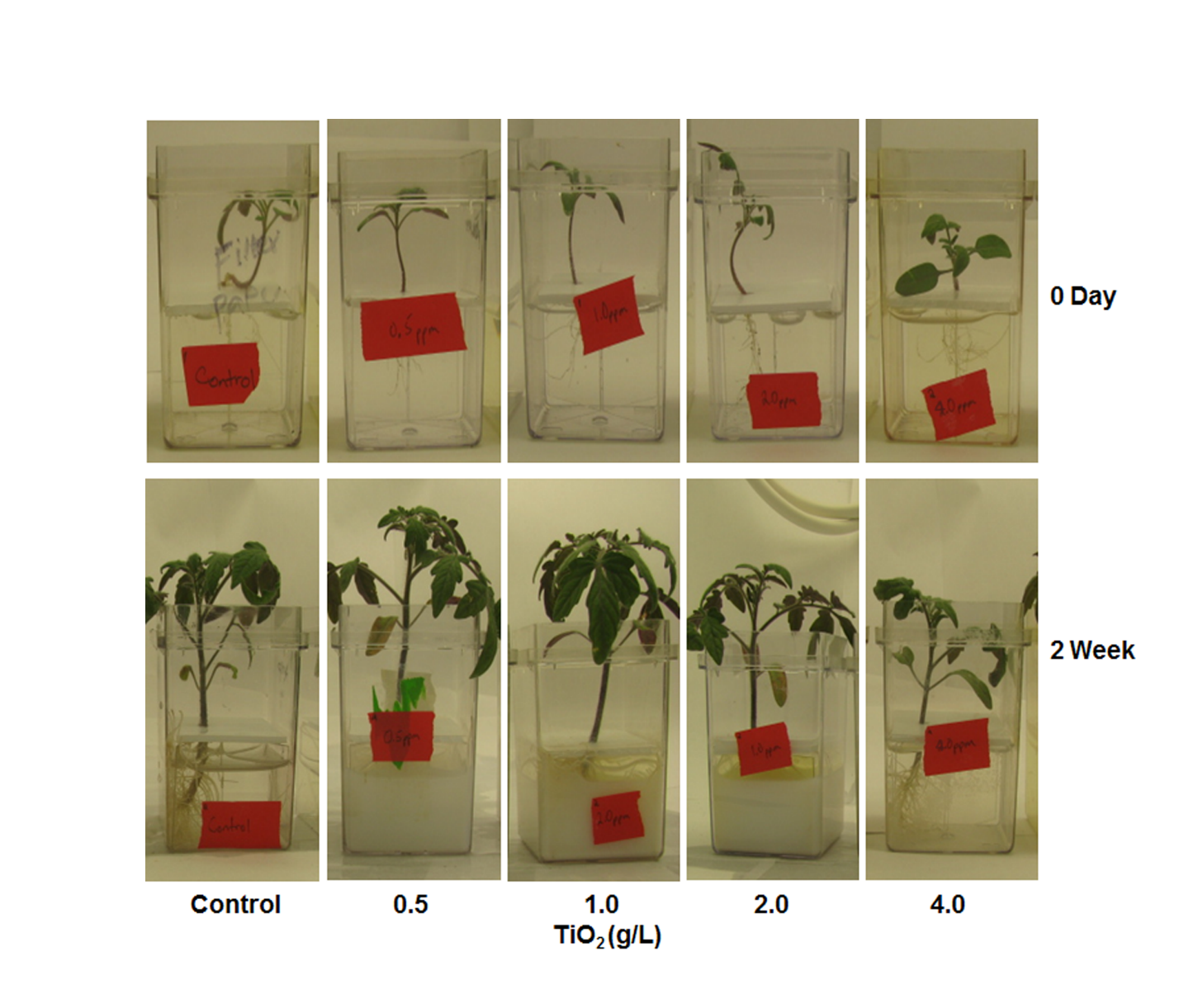


**Figure S2:** Ti content in root tissue. ICP-MS analysis of root samples treated with indicated amount of TiO_2_. Data are presented as ± SEM (n=3) and significant differences between control and treated groups were designated by ** and *** placed on the top of the column that depict (P>0.001 and P>0.0001).


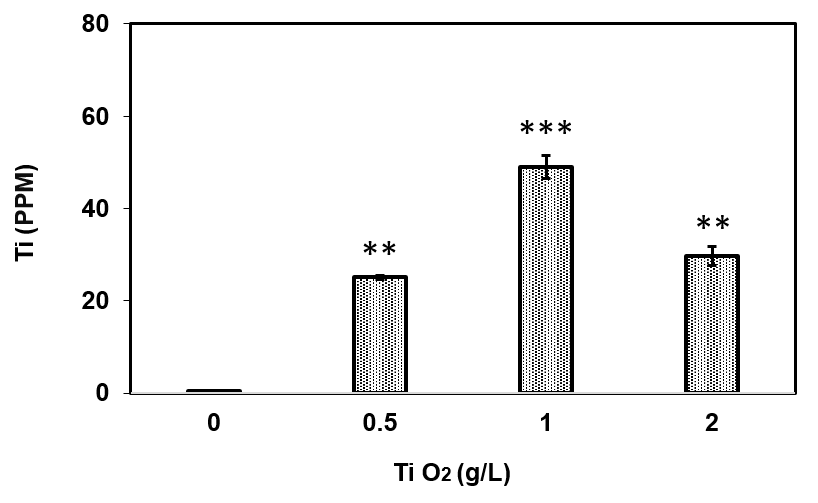

Supplement: TABLE S1 — List of primers used for different genes. [file Data_Sheet_1.docx]
